# Prognostic Significance of CHIP and RIPK3 in Non-Small Cell Lung Cancer

**DOI:** 10.3390/cancers12061496

**Published:** 2020-06-08

**Authors:** Jisup Kim, Joon-Yong Chung, Young Soo Park, Se Jin Jang, Hyeong Ryul Kim, Chang-Min Choi, Joon Seon Song

**Affiliations:** 1 Department of Pathology, Asan Medical Center, University of Ulsan College of Medicine, Seoul 05505, Korea; jspath@amc.seoul.kr (J.K.); youngspark@amc.seoul.kr (Y.S.P.); jangsejin@amc.seoul.kr (S.J.J.); 2 Experimental Pathology Laboratory, Laboratory of Pathology, Center for Cancer Research, National Cancer Institute, National Institutes of Health, Bethesda, MD 20892, USA; chungjo@mail.nih.gov; 3 Center for Cancer Genome Discovery, Asan Medical Center, Asan Institute for Life Sciences, Seoul 05505, Korea; 4 Department of Thoracic and Cardiovascular Surgery, Asan Medical Center, University of Ulsan College of Medicine, Seoul 05505, Korea; drhrkim10@gmail.com; 5 Department of Pulmonology and Critical Care Medicine, Asan Medical Center, University of Ulsan, College of Medicine, Seoul 05505, Korea; 6 Department of Oncology, Asan Medical Center, University of Ulsan, College of Medicine, Seoul 05505, Korea

**Keywords:** necroptosis, carboxyl terminus of Hsp70-interacting protein (CHIP), receptor interacting serine/threonine kinase 3 (RIPK3), prognosis, non-small cell lung cancer

## Abstract

RIPK3 is a key regulator of necroptosis, which plays a double-edged sword role in tumor progression. CHIP is an E3 ubiquitin ligase that regulates necroptosis by degrading RIPK3. Here, we investigated the prognostic value of RIPK3 and CHIP expression in 404 patients with non-small cell lung cancer (NSCLC). Expressions of CHIP and RIPK3 showed opposite correlations with survival. CHIP expression was associated with the longer overall survival (OS), whereas RIPK3 expression was associated with the shorter OS. RIPK3 positivity showed marginal association with shorter OS and disease-free survival (DFS) in adjuvant radiotherapy recipients but not in non-recipients, suggesting that necroptosis may induce radioresistance. In multivariate analysis, CHIP expression was associated with longer OS. Compared with other patients, CHIP(−)/RIPK3(+) patients had shorter OS and DFS. In summary, in patients with NSCLC, the expression of CHIP was an independent favorable prognostic factor while that of RIPK3 was an adverse prognostic factor.

## 1. Introduction

Necroptosis, a caspase-independent programmed cell death modality [1], has recently gained considerable attention as an alternative form of cell death that may overcome the resistance of tumor cells toward apoptosis [2], thereby augmenting antitumor immune responses in cancer patients [3]. Morphologically, necroptosis bears similarities to necrosis such as rupture of the plasma membrane as well as swelling of cells and organelles [4]. Mechanistically, necroptosis bears similarities to apoptosis by sharing a portion of signaling pathways [5]. However, in sharp contrast to apoptosis, necroptosis does not result in the activation of caspases—instead, the amyloid-like signaling complex “necrosome,” which involves the phosphorylated forms of receptor-interacting serine/threonine kinase 1 (RIPK1) and receptor-interacting serine/threonine kinase 3 (RIPK3), is assembled [6]. Upon activation of the necrosome, the downstream effectors of RIPK3 induce the loss of cell and organelle integrity, thereby resulting in the release of endogenous immunomodulatory molecules known as damage-associated molecular patterns (DAMPs) such as high-mobility group box 1 protein and mitochondrial DNA to trigger robust inflammatory responses [7,8,9].

Necroptosis has been implicated in multiple pathophysiological conditions such as ischemia-reperfusion injury, pancreatitis, inflammatory bowel disease, myocardial infarction, stroke, neurodegenerative disorders, sepsis, and chronic obstructive pulmonary disease. Accordingly, small molecules targeting necroptosis have been developed and are being tested in clinical trials. Specifically, GSK2982772 developed by GlaxoSmithKline has been advanced into phase 2 clinical trials for psoriasis, ulcerative colitis, and rheumatoid arthritis [9,10,11,12], and DNL747 developed by Sanofi and Denali Therapeutics is in phase 1 clinical trials for Alzheimer’s disease, amyotrophic lateral sclerosis and multiple sclerosis [12].

Although necroptosis has also been suggested to prevent cancer initiation and progression and to facilitate cancer therapy [13], reports on the significance of necroptosis in cancer progression and metastasis have shown contradicting results. Necroptosis was shown to create an immunosuppressive tumor microenvironment through the recruitment of myeloid-derived suppressor cells or tumor-associated macrophages by increasing the expression of chemokine CXCL1, which may promote tumor growth and progression [14]. Furthermore, necroptosis has been reported to promote cancer metastasis in certain circumstances, as Strilic et al. [15] revealed that circulating tumor cells induce death receptor 6-mediated necroptosis of endothelial cells promoting extravasation and metastasis of tumor cells.

Recently, the carboxyl terminus of Hsp70-interacting protein (CHIP), also known as STIP1 homology and U-box containing protein 1 (STUB1), has been identified as a critical regulator of necroptosis [16]. CHIP is a 34.5-kDa cytoplasmic protein comprising the N-terminal tetratricopeptide (TPR) domain, C-terminal U-box domain, and a central coiled-coil region. As both an E3 ubiquitin ligase and an Hsp70/Hsp90 co-chaperone [17], CHIP implements cellular protein quality control and regulates the stability of its substrate proteins in diverse signaling pathways by inducing ubiquitin-mediated protein degradation via the proteasomal or lysosomal pathways [18].

While RIPK3 phosphorylation (e.g., Ser227 for interaction with MLKL) is a key checkpoint for necroptosis [19], CHIP has been shown to degrade RIPK3 followed by ubiquitylation at Lys55 and Lys 363 in a lysosome-dependent manner, thus negatively regulating RIPK3 expression level [16].

As necroptosis has been reported to display both pro- and anti-tumoral effects during cancer progression and/or metastasis [3,20,21,22], we investigated the prognostic impact of necroptosis in non-small cell lung cancer (NSCLC), which is one of the most common and lethal cancers with a 5-year survival rate of merely 24% [23]. Specifically, we focused on the immunohistochemistry (IHC) expression levels of key regulators of necroptosis (RIPK3 and CHIP).

## 2. Results

### 2.1. Clinicopathologic Characteristics

In total, 404 patients with NSCLC (adenocarcinoma, 247; squamous cell carcinoma, 146; adenosquamous carcinoma, four; large-cell carcinoma, three; sarcomatoid carcinoma, two; carcinoid tumor, two) were included in this retrospective study. The median age of the study population was 63 years old (range, 32–85) and 283 (70.0%) were male. The median follow-up duration was 39.4 months (range, 0.2–122.4). 187 (46.3%) patients received postoperative adjuvant therapy, including 149 (36.9%) patients who received adjuvant chemotherapy (with or without concurrent radiotherapy) and 112 (27.7%) patients who received adjuvant radiotherapy (with or without concurrent chemotherapy). The baseline characteristics of the study population and their associations with CHIP and RIPK3 are presented in Table 1.

### 2.2. Expression of CHIP and RIPK3

**Figure 1:**
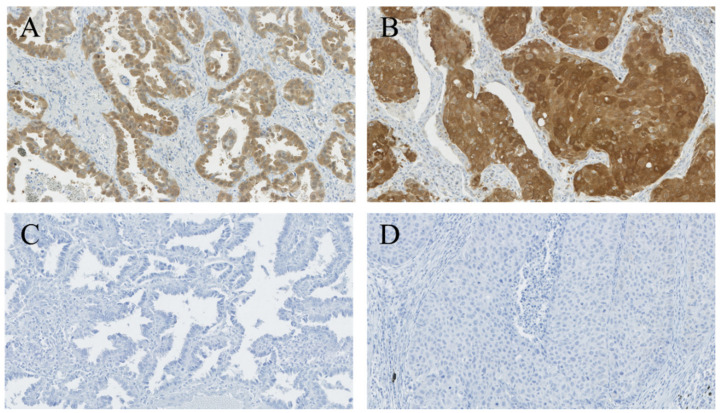
Staining patterns of CHIP. Tumor cells in adenocarcinoma (**A**) and squamous cell carcinoma (**B**) showing highly positive cytoplasmic expression for CHIP. Tumor cells in adenocarcinoma (**C**) and squamous cell carcinoma (**D**) showing immuno-negativity for CHIP. (**A**–**D**, CHIP staining, ×200).

**Figure 2:**
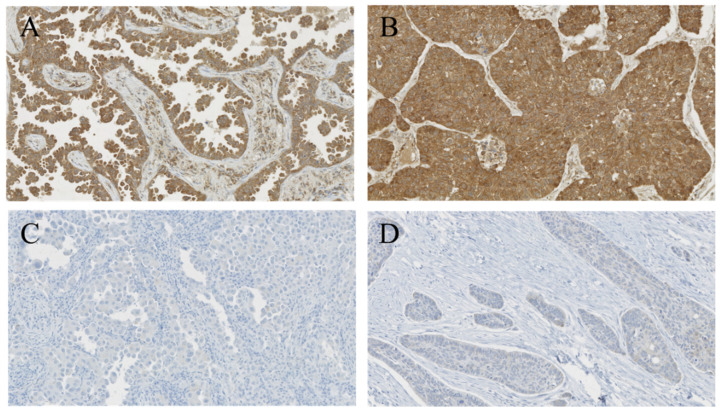
Staining patterns of RIPK3. Tumor cells in adenocarcinoma (**A**) and squamous cell carcinoma (**B**) showing highly positive cytoplasmic expression for RIPK3. Tumor cells in adenocarcinoma (**C**) and squamous cell carcinoma (**D**) showing immuno-negativity for RIPK3. (**A**–**D**, RIPK3 staining, ×200).

In total, 89 (22.0%) cases were highly positive for CHIP (denoted as CHIP(+)) while 315 (78.0%) cases were negative-to-weakly positive (denoted as CHIP(-)). 314 (77.7%) cases were highly positive for RIPK3 (denoted as RIPK3(+)) while 90 (22.3%) cases were negative-to-weakly positive (denoted as RIPK3(−)). In addition, 83 (20.5%) cases were CHIP(+)/RIPK3(+), six (1.5%) were CHIP(+)/RIPK3(−), 231 (57.2%) were CHIP(−)/RIPK3(+), and 84 (20.8%) were CHIP((−))/RIPK3(−). The representative images CHIP and RIPK3 expressions are shown in Figure 1 and Figure 2.

### 2.3. Correlation between CHIP/RIPK3 Expression and Clinicopathologic Parameters

Highly positive CHIP expression was significantly associated with squamous histology (*p* < 0.001), histologic grade 2–3 (*p* = 0.001), and stage II–III with borderline significance (*p* = 0.053) (Table 1). Highly positive RIPK3 expression was significantly associated with stage II–III (*p* < 0.001), grade 2–3 (*p* < 0.001) and postoperative adjuvant therapy (*p* < 0.001) (Table 1). Notably, highly positive CHIP expression was significantly associated with negative-to-weakly positive expression for RIPK3 (*p* < 0.001).

### 2.4. Prognostic Significance of CHIP and RIPK3

**Figure 3:**
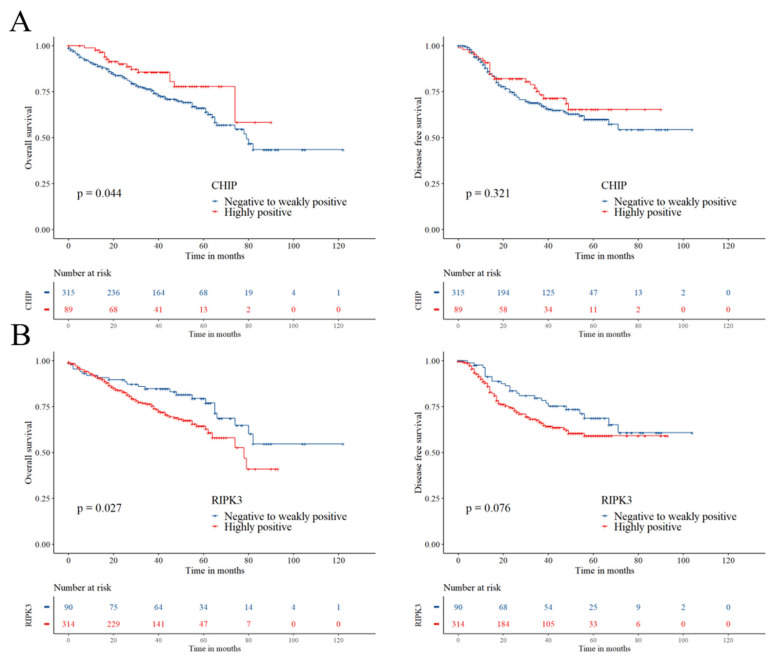
Kaplan–Meier curves for overall survival and disease-free survival according to CHIP (**A**) and RIPK3 (**B**) expression.

In univariate analysis, highly positive CHIP expression was significantly associated with a higher overall survival (OS) rate (hazard ratio (HR) 0.576, 95% confidence interval (CI) 0.334–0.993, log-rank *p* = 0.044) and better disease-free survival (DFS) (HR 0.795, 95% CI 0.504–1.252, log-rank *p* = 0.321), although the latter was not statistically significant (Table 2 and Figure 3). Highly positive RIPK3 expression was significantly associated with lower OS (HR 1.697, 95% CI 1.058–2.721, log-rank *p* = 0.027) and showed a trend toward lower DFS (HR 1.501, 95% CI 0.955–2.361, log-rank *p* = 0.076) (Table 2 and Figure 3).

In subgroup analyses according to adjuvant therapy, highly positive expression of CHIP showed a trend towards better OS (HR 0.526, 95% CI 0.235–1.178, *p* = 0.118) and DFS (HR 0.571, 95% CI 0.288–1.133, *p* = 0.109) in adjuvant chemotherapy recipients (Table 2). Highly positive RIPK3 expression showed a trend towards lower OS in adjuvant chemotherapy non-recipients (HR 1.652, 95% CI 0.958–2.850, *p* = 0.071) (Table 2). Moreover, highly positive RIPK3 expression showed a trend toward lower OS (HR 2.143, 95% CI 0.770–5.969, *p* = 0.145) and lower DFS (HR 2.100, 95% CI 0.829–5.324, *p* = 0.118) in adjuvant radiotherapy recipients, whereas non-recipients showed no notable associations with either prognosis (Table 2).

In subgroup analyses according to histology, highly positive RIPK3 expression was associated with lower OS (HR 1.864, 95% CI 1.035–3.360, *p* = 0.038) and DFS (HR 1.775, 95% CI 1.051–2.999, *p* = 0.032) but not for CHIP in adenocarcinoma (Table S2). Highly positive expression of CHIP showed a trend toward better OS (HR 0.531, 95% CI 0.244–1.154, *p* = 0.110) but not for that of RIPK3 in squamous cell carcinoma (Table S1).

In multivariate analysis, highly positive expression of CHIP was independently associated with better OS (HR 0.500, 95% CI 0.279–0.899, *p* = 0.021), whereas that of RIPK3 did not show a significant association (HR 1.251, 95% CI 0.734–2.130, *p* = 0.410) (Table 3).

### 2.5. Prognostic Significance of the Combinations of CHIP and RIPK3 Expression

To emphasize the prognostic relationship between RIPK3 and CHIP, a potent negative regulator of RIPK3, we divided the patients into two groups in terms of the marker combinations as follows: the first group consisted of patients who were CHIP(−)/RIPK3(+), and the second group consisted of patients who were CHIP(+)/RIPK3(+), CHIP(+)/RIPK3(−), or CHIP(−)/RIPK3(−). The correlation between the two groups and clinicopathologic parameters are shown in Table S2.

**Figure 4:**
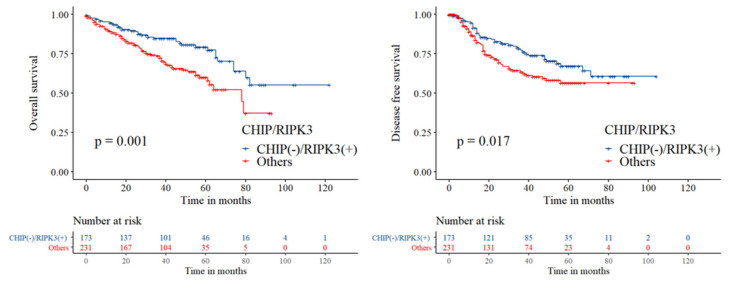
Kaplan–Meier curves for overall survival and disease-free survival between CHIP(−)/RIPK3(+) group and the others including CHIP(+)/RIPK3(+), CHIP(+)/RIPK3(−), and CHIP(−)/RIPK3(−) group. CHIP(+) and RIPK3(+) denote highly positive expression in immunohistochemistry, while CHIP(−) and RIPK3(−) denote negative-to-weakly positive expression.

In univariate analysis, CHIP(−)/RIPK3(+) was associated with both shorter OS (HR 1.999, 95% CI 1.341–2.980, log-rank *p* = 0.001) and shorter DFS (HR 1.566, 95% CI 1.080–2.272, log-rank *p* = 0.017) (Table 2, Figure 4). In multivariate analysis, CHIP(−)/RIPK3(+) was significantly associated with a shorter OS (HR 1.624, 95% CI 1.060–2.487, *p* = 0.026) but not with DFS (HR 1.192, 95% CI 0.804–1.767, *p* = 0.381) (Table 4).

We further compared the survival among the marker combination groups and made three comparisons based on the CHIP(−)/RIPK3(+) group. In univariate analysis, CHIP(−)RIPK3(+) group showed better OS (HR 0.490, 95% CI 0.277–0.866, *p* = 0.014) and CHIP(−)/RIPK3(−) group showed better OS (HR 0.514, 95% CI 0.315–0.840, *p* = 0.008) and DFS (HR 0.592, 95% CI 0.366–0.957, *p* = 0.033) compared with the CHIP(−)/RIPK3(+) group (Table S3). In multivariate analysis, CHIP(+)/RIPK3(+) group showed better OS (HR 0.476, 95% CI 0.260–0.873, *p* = 0.017) compared with the CHIP(−)/RIPK3(+) group (Table S4).

In subgroup analyses according to histology, CHIP(−)/RIPK3(+) was associated with shorter OS (HR 2.096, 95% CI 1.234–3.561, *p* = 0.006) and showed a trend toward shorter OS (HR 1.456, 95% CI 0.936–2.267, *p* = 0.096) in adenocarcinoma (Table S2). CHIP(−)/RIPK3(+) showed a trend toward lower OS (HR 1.686, 95% CI 0.884–3.215, *p* = 0.113) but not for DFS in squamous cell carcinoma (Table S2).

## 3. Discussion

CHIP, an E3 ubiquitin ligase, exhibits both tumorigenic and tumor-suppressive effects depending on its target for ubiquitin-mediated proteasomal or lysosomal degradation [18]. Specifically, in lung cancer, CHIP has been implicated in the proteasomal degradation of oncogenic tyrosine kinase Met receptor, which was demonstrated in an NSCLC xenograft model [24]. In addition, CHIP overexpression reduced the expression of VEGFR2 and the secretion of VEFG in NSCLC cell lines, suggesting the role of CHIP in the degradation of VEGFR, which in turn controls tumor angiogenesis and tumor progression [25,26]. Conversely, a contradictory report revealed that CHIP mediates the ubiquitin-dependent proteasomal degradation of tumor suppressor protein p53 in the human lung cancer cell line H1299 [27]. Considering such a discrepancy in literature which may have been attributed to the limitations of experiments at the cellular or animal level, investigating the prognostic power of CHIP expression in human NSCLC tissue would be meaningful.

Our study is the first study to use IHC to demonstrate the prognostic implication of CHIP expression in NSCLC. There is a similar study carried out by Tingting et al. [25], but the prognostic value of CHIP was studied in association with its mRNA expression levels. We specifically revealed that highly positive cytoplasmic CHIP expression is an independent prognostic factor for OS in patients with NSCLC. Previous reports showed that the prognostic impact of CHIP expression level was different depending on the organ of interest: cytoplasmic CHIP IHC expression was associated with shorter OS in colorectal carcinoma [28] and shorter cancer-specific survival in gallbladder cancer [29]; on the other hand, nuclear CHIP IHC expression was associated with longer cancer-specific survival in breast cancer [30], and cytoplasmic IHC expression was associated with longer OS in head and neck cancer [31].

Considering that CHIP has been reported to suppress necroptosis by targeting RIPK3 for ubiquitin-mediated lysosomal degradation [16], we investigated the relationship between CHIP and RIPK3 IHC expression and found a strong negative correlation between the highly positive expression of CHIP and RIPK3. Furthermore, we found that RIPK3 and CHIP have opposite associations on patient survival, with high expression of RIPK3 being associated with a significantly shorter OS and a tendency of shorter DFS, whereas CHIP showed the opposite associations. Our study reported the prognostic significance of RIPK3 expression in the general population of lung cancer patients regardless of adjuvant therapy in contrast to a previous study that reported the prognostic value of RIPK3 in a specific subgroup of patients who had received adjuvant chemotherapy [32]. In addition, we reclassified the patients into two groups using the marker combinations of CHIP and RIPK3 to further investigate the prognostic impact of CHIP and RIPK3 and found that patients with CHIP(−)/RIPK3(+) had a significantly lower OS and DFS than those with other marker combinations. These results support the previously suggested CHIP-mediated regulatory pathway for necroptosis [16]. Our study uniquely investigated the prognostic significance of CHIP in association with RIPK3, a key regulator of necroptosis. Our results are somewhat contradictory with that of Tingting et al. [25], who reported that CHIP is associated with favorable prognosis NSCLC through the VEGF/VEFGR2 signaling pathway.

In lung cancer, favorable DFS has been associated with RIPK3 IHC expression in lung adenocarcinoma patients who underwent adjuvant chemotherapy [32]. In the report by Chung et al. [32], RIPK3 expression was also associated with better response to cisplatin-based adjuvant chemotherapy as well as high histologic grade. Our study also revealed the association of high expression of RIPK3 with higher histologic grade; however, there was a sharp contrast regarding the prognostic impact of RIPK3 expression because our study revealed a significantly shorter OS in patients with high expressions of RIPK3. Intriguingly, the adverse prognosis in the CHIP(−)/RIPK3(+) group may imply that due to the absence of CHIP, RIPK3 was able to remain intact and activate the necroptosis pathway, which in turn promoted tumor progression; this is in line with the adverse prognosis associated with RIPK3 expression observed in our study.

To investigate the reason for the discrepancy of the prognostic impact of RIPK3 between our cohort and that reported by Chung et al. [32], we performed subgroup analyses according to the histology and the type of adjuvant therapy.

In contrast to the study by Chung et al. [32], RIPK3 was consistently associated with adverse OS and DFS in the adenocarcinoma subgroup and not associated with prognosis in adjuvant chemotherapy recipients. Instead, among patients who received adjuvant radiotherapy, high expression of RIPK3 tended to be associated with shorter OS and DFS, whereas it was not associated with prognosis in radiotherapy non-recipients. Therefore, the opposite prognostic significance of RIPK3 to that previously reported by Chung et al. [32] might be explained by the effect of resistance to radiotherapy, which has been demonstrated to be induced by the expression of necroptotic factors [33].

A previous report showed that deletion of RIPK3 led to reduced expression of the programmed death-ligand 1 (PD–L1) in macrophages as well as increased abundance of B cells and T cells and decreased abundance of myeloid-derived suppressor cells and tumor-associated macrophages [14], suggesting that the suppression of necroptosis by CHIP augmentation might help overcome the immune evasion of tumor cells, an important hallmark of cancer [34].

Hsp90 inhibitors have been recently demonstrated to enhance CHIP-mediated ubiquitylation and degradation of target proteins [35,36]; as such, Hsp90 inhibitors such as 17-AAG and 17-DMAG are under clinical trials as novel targeted therapies [37]. Therefore, enhancing CHIP-mediated ubiquitylation by HSP90 inhibitors may also show promise in NSCLC patients, possibly by potentiating radiosensitivity and anti-tumor immunity to improve survival.

Necroptosis, a novel form of cell death, has gained considerable attention for targeting cancer cells due its double-edged sword effect in cancer through its protumoral and antitumoral effects [3,20,21,22]. Our study is the first to demonstrate the opposing prognostic relationship between RIPK3, a crucial inducer of necroptosis, and CHIP, a potent negative regulator of RIPK3. The adverse prognosis of RIPK3 as well as the favorable prognosis of CHIP revealed in this study emphasize the protumoral effect of necroptosis in NSCLC. In addition,, the prognostic significance may improve the management of patients with NSCLC by appropriate patient selection through individualized risk stratification.

## 4. Materials and Methods

### 4.1. Tumor Samples

The study was carried out with tissues collected from 404 NSCLC patients who underwent complete surgical resection at Asan Medical Center between 2000 and 2010. Patients who received neoadjuvant chemotherapy or those diagnosed with distant metastasis at initial presentation were excluded. Clinicopathological characteristics including survival data were retrospectively collected by review of medical records. Tumors were staged according to the 8th edition of the American Joint Committee on Cancer tumor-node-metastasis staging system, and histologic grading and subtyping followed the World Health Organization’s guidelines. Tissue microarrays with 2 mm-diameter cores were constructed from representative tumor sections using formalin-fixed paraffin-embedded blocks. This study was approved by the institutional review board of Asan Medical Center (2019–1215, Seoul, Republic of Korea) and conformed to the tenets of the Declaration of Helsinki.

### 4.2. Immunohistochemistry

IHC for CHIP1 and RIPK3 was performed using monoclonal mouse anti-RIPK3 antibody (1:500; R&D system, Minneapolis, MN, USA, clone #780115) and rabbit anti-CHIP antibody (1:100; Cell Signaling Technology, Beverly, MA, USA) on tissue microarray samples. In brief, following deparaffinization and dehydration, heat-induced antigen retrieval was performed for 20 min in an antigen retrieval buffer of pH 9.0 (for RIPK3) or pH 6.0 (for CHIP) (Dako, Carpinteria, CA) using a steam pressure cooker (Pascal, Dako). The antigen-antibody reaction was detected with EnVision+ Dual Link System-HRP (Dako) and visualized with 3, 3′-Diaminobenzidine (DAB+; Dako). In the negative control section, the primary antibody was omitted. The positive control was gastric cancer tissue for RIPK3 and breast cancer tissue for CHIP. Tissue sections were lightly counterstained with hematoxylin and then examined by light microscopy.

The evaluation of immunostaining was done using a computer-assisted image analyzing software version 4.5.1.324 (Visiopharm, Hoersholm, Denmark). In brief, stained slides were scanned by NanoZoomer 2.0 HT (Hamamatsu Photonics, Hamamatsu City, Japan) at 20× objective magnification (0.5 μm resolution). Captured digital images were then imported into Visiopharm software. Each core was imported separately using the tissue microarray workflow of the program. For image analysis, a number of steps are typically required. First, the transformation of an image from one form to another (image processing) is done to enhance image structures of relevance for subsequent image segmentation. After training the system by digitally “painting” examples of the image, areas are defined which is named segmentation. The mean intensity of DAB of each defined image is used for the quantification of the expressions. Immunoreactivity was assessed using the semi-quantitative H-score (range 0–300), which is derived by summing of each staining intensity (0–3) multiplied by the percentage (0–100) of immunoreactive cells of that intensity.

### 4.3. Statistical Analysis

Baseline descriptive statistics were expressed as median (minimum–maximum) for continuous variables and frequency (percentage) for categorical variables. Chi-squared test and Fisher’s exact test were used for comparison of categorical variables.

In order to identify the association of the immunoreactivity of necroptosis-associated markers with clinicopathological features and survival, the immunoreactivity was categorized into negative-to-weakly positive and highly positive using optimal cut-off values determined by maximally selected log-rank statistics by Hothorn and Lausen [38]. The optimal cut-off values were 241.7 for CHIP and 189.2 for RIPK3.

DFS was assessed as the interval between the date of surgery and the date of documentation of either locoregional recurrence or distant metastasis, whichever occurred first. OS was assessed as the interval between the date of surgery and the date of death from any cause or last follow-up.

Survival curves were calculated using the Kaplan–Meier product-limit method, and DFS and OS were compared using the log-rank test as well as univariate Cox proportional-hazards regression model. To adjust for the clinicopathologic variables across immunoreactivity or clinically important factors, multivariate Cox proportional-hazards regression model was utilized. All reported *p* values are two-tailed, and those <0.05 were considered statistically significant.

Statistical analysis was carried out using IBM SPSS Statistics for Windows, version 21 (IBM Corp., Armonk, NY, USA) and the R software version 3.5.3 (R Foundation for Statistical Computing, Vienna, Austria) with the “maxstat” package.

## 5. Conclusions

We showed that RIPK3 is an adverse prognosticator for OS whereas CHIP is an independent favorable prognosticator for OS in NSCLC. Furthermore, we have demonstrated that the CHIP(−)/RIPK3(+) group was significantly associated with adverse OS. Our current findings not only shed light on the prognostic implications of the positive and negative regulators of necroptosis but also help to risk-stratify patients for individualized management in NSCLC.

## Figures and Tables

**Table 1:** Clinicopathologic features of the study population and their associations with the expression of CHIP and RIPK3.

Clinicopathologic Variables	All (*n* = 404)	CHIP (-) (*n* = 315)	CHIP (+) (*n* = 89)	*p*	RIPK3 (−) (*n* = 90)	RIPK3 (+) (*n* = 314)	*p*
**Age**							
<65 yrs	245 (60.6%)	193 (61.3%)	52 (58.4%)	0.628	59 (65.6%)	186 (59.2%)	0.279
≥65 yrs	159 (39.4%)	122 (38.7%)	37 (41.6%)		31 (34.4%)	128 (40.8%)	
**Gender**							
Male	283 (70.0%)	218 (69.2%)	65 (73.0%)	0.486	64 (71.1%)	219 (69.7%)	0.803
Female	121 (30.0%)	97 (30.8%)	24 (27.0%)		26 (28.9%)	95 (30.3%)	
**Smoking**							
No smoking history	146 (36.1%)	119 (37.8%)	27 (30.3%)	0.197	32 (35.6%)	114 (36.3%)	0.896
Smoking history	258 (63.9%)	196 (62.2%)	62 (69.7%)		58 (64.4%)	200 (63.7%)	
**Operation type**							
Lobectomy	348 (86.1%)	273 (86.7%)	75 (84.3%)	0.412	81 (90.0%)	267 (85.0%)	0.576
Bilobectomy	26 (6.4%)	20 (6.3%)	6 6.7%)		3 (3.3%)	23 (7.3%)	
Pneumonectomy	25 (6.2%)	17 (5.4%)	8 (9.0%)		5 (5.6%)	20 (6.4%)	
Others ^a^	5 (1.2%)	5 (1.6%)	0 (0%)		1 (1.1%)	4 (1.3%)	
**Stage**							
I	177 (43.8%)	146 (46.3%)	31 (34.8%)	0.053	63 (70.0%)	114 (36.3%)	<0.001
II–III	227 (56.2%)	169 (53.7%)	58 (65.2%)		27 (30.0%)	200 (63.7%)	
**Histology**							
Adenocarcinoma	247 (61.1%)	209 (66.3%)	38 (42.7%)	<0.001	64 (71.1%)	183 (58.3%)	0.077
Squamous cell carcinoma	146 (36.1%)	97 (30.8%)	49 (55.1%)		24 (26.7%)	122 (38.9%)	
Other types ^b^	11 (2.7%)	9 (2.9%)	2 (2.2%)		2 (2.2%)	9 (2.9%)	
**Grade**							
1	86 (21.9%)	78 (25.4%)	8 (9.3%)	0.001	31 (35.6%)	55 (18.0%)	<0.001
2–3	307 (76.0%)	229 (74.6%)	78 (90.7%)		56 (64.4%)	251 (82.0%)	
**Adjuvant therapy**							
Not performed	217 (53.7%)	172 (54.6%)	45 (50.6%)	0.500	70 (77.8%)	147 (46.8%)	<0.001
Performed	187 (46.3%)	143 (45.4%)	44 (49.4%)		20 (22.2%)	167 (53.2%)	
**RIPK3**							
Negative-to-weakly positive	90 (22.3%)	84 (26.7%)	6 (6.7%)	<0.001	N/A	N/A	N/A
Highly positive	314 (77.7%)	231 (73.3%)	83 (93.3%)		N/A	N/A	

CHIP(+) and RIPK3(+) denote highly positive expression by the immunohistochemistry, while CHIP(−) and RIPK3(−) denote negative-to-weakly positive expression. ^a^ Other operation types include two lobectomies with wedge resection, two segmentectomies, and one wedge resection. ^b^ Other histology includes four adenosquamous carcinomas, three large-cell carcinomas, two sarcomatoid carcinomas, and two carcinoid tumors.

**Table 2:** Univariate Cox regression analysis for overall survival and disease-free survival.

Variable	Overall Survival		Disease-Free Survival	
	HR (95% CI)	*p*	HR (95% CI)	*p*
**General population**				
Age ≥ 65 (vs. <65)	1.640 (1.134–2.372)	**0.009**	1.052 (0.729–1.518)	**0.787**
Male (vs. female)	1.678 (1.087–2.589)	**0.019**	1.137 (0.771–1.676)	**0.516**
Smoking history (vs. no smoking history)	1.611 (1.076–2.414)	**0.021**	0.949 (0.658–1.368)	**0.779**
**Operation type**				
Lobectomy	1		1	
Bilobectomy	1.649 (0.857–3.174)	**0.134**	1.226 (0.620–2.423)	**0.557**
Pneumonectomy	2.123 (1.065–4.232)	**0.032**	0.911 (0.400–2.075)	**0.825**
Others ^a^	2.343 (1.015–5.406)	**0.046**	2.088 (0.291–14.994)	**0.464**
Adjuvant therapy (+ vs. −)	2.322 (1.565–3.445)	**<0.001**	2.460 (1.696–3.567)	**<0.001**
**Histology**				
Adenocarcinoma	1		1	
Squamous cell carcinoma (vs. adenocarcinoma)	1.154 (0.781–1.708)	**0.472**	0.603 (0.400–0.910)	**0.016**
Other types ^b^ (vs. adenocarcinoma)	5.064 (2.309–11.107)	**<0.001**	2.199 (0.805–6.003)	**0.124**
Stage II–III (vs. Stage I)	4.758 (2.952–7.669)	**<0.001**	2.257 (1.539–3.309)	**<0.001**
Grade 2–3 (vs. Grade 1)	3.127 (1.743–5.609)	**<0.001**	1.719 (1.077–2.745)	**0.023**
CHIP expression (+ vs. −)	0.576 (0.334–0.993)	**0.047**	0.795 (0.504–1.252)	**0.322**
RIPK3 expression (+ vs. −)	1.697 (1.058–2.721)	**0.028**	1.501 (0.955–2.361)	**0.078**
CHIP(-)/RIPK3(+) (vs. Others ^c^)	1.999 (1.341–2.980)	**0.001**	1.566 (1.080–2.272)	**0.018**
**Adjuvant chemotherapy recipients**				
CHIP expression (+ vs. −)	0.526 (0.235–1.178)	**0.118**	0.571 (0.288–1.133)	**0.109**
RIPK3 expression (+ vs. −)	1.219 (0.436–3.409)	**0.706**	1.310 (0.521–3.297)	**0.566**
**Adjuvant chemotherapy non-recipients**				
CHIP expression (+ vs. −)	0.584 (0.279–1.225)	**0.155**	0.968 (0.527–1.780)	**0.918**
RIPK3 expression (+ vs. −)	1.652 (0.958–2.850)	**0.071**	1.301 (0.757–2.236)	**0.340**
**Adjuvant radiotherapy recipients**				
CHIP expression (+ vs. −)	0.555 (0.236–1.305)	**0.177**	0.977 (0.473–2.015)	**0.949**
RIPK3 expression (+ vs. −)	2.143 (0.770–5.969)	**0.145**	2.100 (0.829–5.324)	**0.118**
**Adjuvant radiotherapy non-recipients**				
CHIP expression (+ vs. −)	0.624 (0.307–1.267)	**0.192**	0.795 (0.442–1.428)	**0.442**
RIPK3 expression (+ vs. −)	1.322 (0.756–2.310)	**0.327**	1.199 (0.703–2.045)	**0.506**

CHIP(+) and RIPK3(+) denote highly positive expression in immunohistochemistry, while CHIP(−) and RIPK3(−) denote negative-to-weakly positive expression. ^a^ Other operation types include two lobectomies with wedge resection, two segmentectomies, and one wedge resection. ^b^ Other histology includes four adenosquamous carcinomas, three large-cell carcinomas, two sarcomatoid carcinomas, and two carcinoid tumors. ^c^ Others consisted of 83 CHIP(+)/RIPK3(+), six CHIP(+)/RIPK3(−), and 84 CHIP(−)/RIPK3(−) cases. CI, confidence interval; HR, hazard ratio.

**Table 3:** Multivariate Cox regression analysis on overall survival according to CHIP and RIPK3 expression.

Variable	Overall Survival	
	HR (95% CI)	*p*
Age ≥ 65 (vs. <65)	1.445 (0.926–2.255)	**0.105**
Male (vs. female)	1.435 (0.673–3.057)	**0.350**
Smoking history (vs. no smoking history)	0.992 (0.490–2.009)	**0.982**
**Operation type**		
Lobectomy	1	
Bilobectomy	1.036 (0.498–2.153)	**0.926**
Pneumonectomy	1.432 (0.669–3.068)	**0.355**
Others ^a^	7.375 (2.232–24.370)	**0.001**
Adjuvant therapy (+ vs. −)	0.526 (0.291–0.949)	**0.033**
**Histology**		
Adenocarcinoma	1	
Squamous cell carcinoma (vs. adenocarcinoma)	0.609 (0.366–1.012)	**0.056**
Other types ^b^ (vs. adenocarcinoma)	4.836 (1.603–14.590)	**0.005**
Stage II–III (vs. Stage I)	7.101 (3.562–14.156)	**<0.001**
Grade 2–3 (vs. Grade 1)	2.245 (1.176–4.288)	**0.014**
CHIP expression (+ vs. −)	0.500 (0.279–0.899)	**0.021**
RIPK3 expression (+ vs. −)	1.251 (0.734–2.130)	**0.410**

CHIP(+) and RIPK3(+) denote highly positive expression in immunohistochemistry, while CHIP(−) and RIPK3(−) denote negative-to-weakly positive expression. ^a^ Other operation types include two lobectomies with wedge resection, two segmentectomies, and one wedge resection. ^b^ Other histology includes four adenosquamous carcinomas, three large-cell carcinomas, two sarcomatoid carcinomas, and two carcinoid tumors. CI, confidence interval; HR, hazard ratio.

**Table 4:** Multivariate Cox regression analysis on overall survival and disease-free survival according to the groups based on the combination of CHIP and RIPK3 expression.

Variable	Overall Survival		Disease-Free Survival	
	HR (95% CI)	*p*	HR (95% CI)	*p*
Age ≥ 65 (vs. <65)	1.407 (0.904–2.190)	**0.131**	–	–
Male (vs. female)	1.512 (0.714–3.204)	**0.280**	–	–
Smoking history (vs. no smoking history)	0.984 (0.487–1.988)	**0.964**	–	–
**Operation type**				
Lobectomy	1		1	
Bilobectomy	1.042 (0.501–2.168)	**0.912**	1.409 (0.667–2.977)	**0.369**
Pneumonectomy	1.437 (0.671–3.078)	**0.351**	1.098 (0.412–2.926)	**0.851**
Others ^a^	8.085 (2.527–25.873)	**<0.001**	2.957 (0.399–21.910)	**0.289**
Adjuvant therapy (+ vs. −)	0.516 (0.286–0.931)	**0.028**	1.538 (0.767–3.087)	**0.225**
**Histology**				
Adenocarcinoma	1		1	
Squamous cell carcinoma (vs. adenocarcinoma)	0.575 (0.348–0.948)	**0.030**	0.385 (0.235–0.632)	**<0.001**
Other types ^b^ (vs. adenocarcinoma)	5.112 (1.737–15.039)	**0.003**	3.655 (1.038–12.872)	**0.044**
Stage II–III (vs. Stage I)	6.820 (3.435–13.539)	**<0.001**	1.683 (0.819–3.461)	**0.157**
Grade 2–3 (vs. Grade 1)	2.198 (1.150–4.201)	**0.017**	1.678 (1.011–2.784)	**0.045**
CHIP(−)/RIPK3(+) (vs. Others ^c^)	1.624 (1.060–2.487)	**0.026**	1.192 (0.804–1.767)	**0.381**

^a^ Other operation types include two lobectomies with wedge resection, two segmentectomies, and one wedge resection. ^b^ Other histology includes four adenosquamous carcinomas, three large-cell carcinomas, two sarcomatoid carcinoma, and two carcinoid tumors. ^c^ Others consisted of 83 CHIP(+)/RIPK3(+), six CHIP(+)/RIPK3(−), and 84 CHIP(−)/RIPK3(−) cases. CI, confidence interval; HR, hazard ratio. CHIP(+) and RIPK3(+) denote highly positive expression in immunohistochemistry, while CHIP(−) and RIPK3(−) denote negative-to-weakly positive expression.

## Supplementary Materials

The following are available online at https://www.mdpi.com/2072-6694/12/6/1496/s1, Table S1: Univariate Cox regression analysis for overall survival and disease-free survival in subgroups according to histology, Table S2: Clinicopathologic features of the study population and their associations with the combination groups according to CHIP and RIPK3, Table S3: Univariate Cox regression analysis for overall survival and disease-free survival according to the four marker combination groups by CHIP and RIPK3 expression, Table S4: Multivariate Cox regression analysis on overall survival and disease-free survival according to the four marker combination groups by CHIP and RIPK3 expression.
