# Supplementary material for: Prognostic Significance of CHIP and RIPK3 in Non-Small Cell Lung Cancer
Source: Cancers (Basel). 2020 Jun 8;12(6):1496. doi: 10.3390/cancers12061496 (PMC7352347; doi:10.3390/cancers12061496)
Supplement: Supplementary file 1 [file cancers-12-01496-s001.pdf]

Supplementary materials

Prognostic Significance of CHIP and RIPK3 in Non-Small Cell Lung Cancer

Jisup Kim [1], Joon-Yong Chung [2], Young Soo Park [1], Se Jin Jang [1,3], Hyeong Ryul Kim [4], Chang-Min Choi [5,6,*] and Joon Seon Song [1,*]

[1] Department of Pathology, Asan Medical Center, University of Ulsan College of Medicine, Seoul 05505, Korea; jspath@amc.seoul.kr (J.K.), youngspark@amc.seoul.kr (Y.S.P.), jangsejin@amc.seoul.kr (S.J.J.)
[2] Experimental Pathology Laboratory, Laboratory of Pathology, Center for Cancer Research, National Cancer Institute, National Institutes of Health, Bethesda, MD 20892, USA; chungjo@mail.nih.gov
[3] Center for Cancer Genome Discovery, Asan Medical Center, Asan Institute for Life Sciences, Seoul 05505, Korea
[4] Department of Thoracic and Cardiovascular Surgery, Asan Medical Center, University of Ulsan College of Medicine, Seoul 05505, Korea; drhrkim10@gmail.com
[5] Department of Pulmonology and Critical Care Medicine, Asan Medical Center, University of Ulsan, College of Medicine, Seoul 05505, Korea
[6] Department of Oncology, Asan Medical Center, University of Ulsan, College of Medicine, Seoul 05505, Korea
* Correspondence: ccm9607@gmail.com (C.-M.C.); songjs@amc.seoul.kr (J.S.S.); Tel.: +82-2-3010-5902 (C.-M.C.); +82-2-3010-4548 (J.S.S.)

Table S1. Univariate Cox regression analysis for overall survival and disease-free survival in subgroups according to histology.

Variable	Overall survival		Disease-free survival	
	HR (95% CI)	*p*	HR (95% CI)	*p*
**Adenocarcinoma**				
CHIP expression (+ vs. -)	0.669 (0.305–1.467)	0.316	1.176 (0.673–2.056)	0.568
RIPK3 expression (+ vs. -)	1.864 (1.035–3.360)	**0.038**	1.775 (1.051–2.999)	**0.032**
CHIP(-)/RIPK3(+) (vs. Others[c])	2.096 (1.234–3.561)	**0.006**	1.456 (0.936–2.267)	0.096
**Squamous cell carcinoma**				
CHIP expression (+ vs. -)	0.531 (0.244–1.154)	0.110	0.689 (0.306–1.551)	0.368
RIPK3 expression (+ vs. -)	1.298 (0.556–3.030)	0.547	1.142 (0.447–2.920)	0.781
CHIP(-)/RIPK3(+) (vs. Others[a])	1.686 (0.884–3.215)	0.113	0.689 (0.306–1.551)	0.368

CHIP(+) and RIPK3(+) denote highly positive expression in immunohistochemistry, while CHIP(-) and RIPK3(-) denote negative-to-weakly positive expression. [a] Others consisted of 83 CHIP(+)/RIPK3(+), 6 CHIP(+)/RIPK3(-), and 84 CHIP(-)/RIPK3(-) cases. CI, confidence interval; HR, hazard ratio.

Table S2. Clinicopathologic features of the study population and their associations with the combination groups according to CHIP and RIPK3.

Clinicopathologic variables	All (*n*=404)	CHIP(-) /RIPK3(+) (*n*=231)	Others[c] (*n*=173)	*p*
Age				
< 65 yrs	245 (60.6%)	105 (60.7%)	140 (60.6%)	0.986
≥ 65 yrs	159 (39.4%)	68 (39.3%)	91 (39.4%)	
Gender				
Male	283 (70.0%)	157 (68.0%)	126 (72.8%)	0.291
Female	121 (30.0%)	74 (32.0%)	47 (27.2%)	
Smoking				
No smoking history	146 (36.1%)	89 (38.5%)	57 (32.9%)	0.248
Smoking history	258 (63.9%)	142 (61.5%)	116 (67.1%)	
Operation type				
Lobectomy	348 (86.1%)	198 (85.7%)	150 (86.7%)	0.461
Bilobectomy	26 (6.4%)	17 (7.4%)	9 (5.2%)	
Pneumonectomy	25 (6.2%)	12 (5.2%)	13 (7.5%)	
Others[a]	5 (1.2%)	4 (1.7%)	1 (0.6%)	
Stage				
I	177 (43.8%)	86 (37.2%)	91 (52.6%)	0.002
II–III	227 (56.2%)	145 (62.8%)	82 (47.4%)	
Histology				
Adenocarcinoma	247 (61.1%)	147 (63.6%)	100 (57.8%)	0.398
Squamous cell carcinoma	146 (36.1%)	77 (33.3%)	69 (39.9%)	
Other types[b]	11 (2.7%)	7 (3.0%)	4 (2.3%)	
Grade				
1	86 (21.9%)	48 (21.2%)	38 (22.8%)	0.719
2–3	307 (76.0%)	178 (78.8%)	129 (77.2%)	
Adjuvant therapy				
Not performed	217 (53.7%)	105 (45.5%)	112 (64.7%)	<0.001
Performed	187 (46.3%)	126 (54.5%)	61 (35.3%)	

CHIP(+) and RIPK3(+) denote highly positive expression in immunohistochemistry, while CHIP(-) and RIPK3(-) denote negative-to-weakly positive expression. [a]Other operation types include 2 lobectomies with wedge resection, 2 segmentectomies, and 1 wedge resection. [b]Other histology includes 4 adenosquamous carcinomas, 3 large-cell carcinomas, 2 sarcomatoid carcinomas, and 2 carcinoid tumors. [c]Others consisted of 83 CHIP(+)/RIPK3(+), 6 CHIP(+)/RIPK3(-), and 84 CHIP(-)/RIPK3(-) cases.

Table S3. Univariate Cox regression analysis for overall survival and disease-free survival according to the four marker combination groups by CHIP and RIPK3 expression.

Variable	Overall survival		Disease-free survival	
	HR (95% CI)	*p*	HR (95% CI)	*p*
CHIP(-)/RIPK3(+)	1	-	1	-
CHIP(+)/RIPK3(-)	0.397 (0.055–2.856)	0.359	0.777 (0.191–3.166)	0.725
CHIP(+)/RIPK3(+)	0.490 (0.277–0.866)	**0.014**	0.682 (0.420–1.106)	0.121
CHIP(-)/RIPK3(-)	0.514 (0.315–0.840)	**0.008**	0.592 (0.366–0.957)	**0.033**

CHIP(+) and RIPK3(+) denote highly positive expression in immunohistochemistry, while CHIP(-) and RIPK3(-) denote negative-to-weakly positive expression.

Table S4. Multivariate Cox regression analysis on overall survival and disease-free survival according to the four marker combination groups by CHIP and RIPK3 expression.

Variable	Overall survival		Disease-free survival	
	HR (95% CI)	*p*	HR (95% CI)	*p*
Age ≥ 65 (vs. < 65)	1.457 (0.932–2.278)	0.098	-	-
Male (vs. female)	1.482 (0.695–3.161)	0.309	-	-
Smoking history (vs. no smoking history)	0.975 (0.482–1.971)	0.944	-	-
Operation type				
Lobectomy	1		1	-
Bilobectomy	1.047 (0.503–2.178)	0.903	1.437 (0.678–3.048)	0.344
Pneumonectomy	1.460 (0.680–3.135)	0.332	1.121 (0.419–2.999)	0.820
Others[a]	7.544 (2.291–24.846)	**0.001**	3.012 (0.406–22.332)	0.281
Stage II–III (vs. Stage I)	7.115 (3.565–14.198)	**< 0.001**	1.674 (0.813–3.449)	0.162
Histology				
Adenocarcinoma	1	-	1	-
Squamous cell carcinoma (vs. adenocarcinoma)	0.600 (0.361–0.997)	**0.049**	0.371 (0.223–0.619)	**< 0.001**
Other types[b] (vs. adenocarcinoma)	4.827 (1.606–14.505)	**0.005**	3.641 (1.033–12.829)	**0.044**
Grade 2–3 (vs. Grade 1)	2.246 (1.176–4.291)	**0.014**	1.680 (1.011–2.793)	**0.045**
Adjuvant therapy (+ vs. -)	0.521 (0.288–0.941)	**0.031**	1.528 (0.760–3.070)	0.234
Marker combination groups				
CHIP(-)/RIPK3(+)	1	**-**	1	-
CHIP(+)/RIPK3(-)	0.900 (0.119–6.782)	0.919	1.289(0.305–5.441)	0.730
CHIP(+)/RIPK3(+)	0.476 (0.260–0.873)	**0.017**	0.869(0.521–1.450)	0.591
CHIP(-)/RIPK3(-)	0.766 (0.444–1.323)	0.339	0.785 (0.471–1.310)	0.355

CHIP(+) and RIPK3(+) denote highly positive expression in immunohistochemistry, while CHIP(-) and RIPK3(-) denote negative-to-weakly positive expression. [a]Other operation types include 2 lobectomies with wedge resection, 2 segmentectomies, and 1 wedge resection. [b]Other histology includes 4 adenosquamous carcinomas, 3 large-cell carcinomas, 2 sarcomatoid carcinoma, and 2 carcinoid tumors. CI, confidence interval; HR, hazard ratio.
